# The deficiency of galectin-3 in stromal cells leads to enhanced tumor growth and bone marrow metastasis

**DOI:** 10.1186/s12885-016-2679-1

**Published:** 2016-08-15

**Authors:** Jonathas Xavier Pereira, Maria Carolina Braga Azeredo, Felipe Sá Martins, Roger Chammas, Felipe Leite Oliveira, Sofia Nascimento Santos, Emerson Soares Bernardes, Márcia Cury El-Cheikh

**Affiliations:** 1 Programa de Pós-Graduação em Anatomia Patológica, Hospital Clementino Fraga Filho, UFRJ, Rio de Janeiro, Brazil; 2 Programa de Pós-Graduação em Ciências Morfológicas, ICB, UFRJ, Rio de Janeiro, Brazil; 3 Universidade Federal do Rio de Janeiro, Rio de Janeiro, Brazil; 4 Laboratório de Oncologia Experimental e Instituto do Câncer do Estado de São Paulo, Faculdade de Medicina, São Paulo, Brazil; 5 Laboratório de Proliferação e Diferenciação Celular, ICB, UFRJ, Rio de Janeiro, RJ Brazil; 6 Centro de Radiofarmácia, Instituto de Pesquisas Energéticas e Nucleares (IPEN), São Paulo, Brazil; 7 Cidade Universitária, Ilha do Fundão, Instituto de Ciências Biomédicas, CCS, Av. Carlos Chagas Filho, 393. Bloco F, CEP. 21941-902 Rio Janeiro, RJ Brazil

**Keywords:** 4T1 breast carcinoma, Galectin-3, Bone marrow metastasis, CXCR4/CXCL12 axis

## Abstract

**Background:**

Galectin-3 is a multifunctional β-galactoside-binding lectin that once synthesized, is expressed in the nucleus, cytoplasm, cell surface and in the extracellular environment. Because of its unique structure, galectin-3 can oligomerize forming lattice upon binding to multivalent oligossacharides and influence several pathologic events such as tumorigenesis, invasion and metastasis.

**Methods:**

In our study, balb/c Lgals3+/+ and Lgals3−/− female mice were inoculated in the fourth mammary fat pad with 4T1 breast cancer cell line. The primary tumor, inguinal lymph nodes and iliac bone marrow were evaluated 15, 21 and 28 days post-injection. The primary tumor growth was evaluated by measuring the external diameter, internal growth by ultrasound and weight of the excised tumor. The presence of cancer cells in the draining lymph nodes and iliac crest bone marrow were performed by immunohistochemistry, PCR and clonogenic metastatic assay.

**Results:**

In this study we demonstrated that the deletion of galectin-3 in the host affected drastically the in vivo growth rate of 4T1 tumors. The primary tumors in Lgals3−/− mice displayed a higher proliferative rate (*p* < 0,05), an increased necrotic area (*p* < 0,01) and new blood vessels with a wider lumen in comparison with tumors from Lgals3+/+ mice (*P* < 0,05). Moreover, we detected a higher number of 4T1-derived metastatic colonies in the lymph nodes and the bone marrow of Lgals3−/− mice (*p* < 0,05). Additionally, healthy Lgals3−/− control mice presented an altered spatial distribution of CXCL12 in the bone marrow, which may explain at least in part the initial colonization of this organ in Lgals3−/− injected with 4T1 cells.

**Conclusions:**

Taken together, our results demonstrate for the first time that the absence of galectin-3 in the host microenvironment favors the growth of the primary tumors, the metastatic spread to the inguinal lymph nodes and bone marrow colonization by metastatic 4T1 tumor cells.

**Electronic supplementary material:**

The online version of this article (doi:10.1186/s12885-016-2679-1) contains supplementary material, which is available to authorized users.

## Background

Galectin-3, a glycan-binding protein, is one the most studied galectins due to its peculiar structure presenting an N-terminal non lectin domain and a C-terminal carbohydrate recognition domain with affinity for β-galactosides (CRD), that facilitates its dimerization and formation of a bridge or lattice between cells and extracellular compartment [1–4]. Once synthesized, galectin-3 shuttles between cytoplasm and nucleus, and also is secreted to the cell surface and into the biological fluids [2]. Thus, galectin-3 can act as an adhesion molecule controlling crucial cellular events as migration, cell proliferation, differentiation and apoptosis [4].

Galectin-3 plays an important role in processes that fuel the tumor growth and metastasis [3–6]. Exogenous galectin-3 enhances the endothelial cell mobility in vitro and promotes new capillaries formation in vivo [5]. In several tumors, it is highly expressed and its concentrations are markedly increased in the patient’s serum [6]. Galectin-3 and its glycoconjugate ligands prolong the tumor cell survival in the circulation by promoting tumor cell homotypic aggregation, thus facilitating their dissemination and preventing anoikis [6, 7].

However, galectin-3 is generated not only by tumor, but also by peri tumoral inflammatory and stromal cells [8], indicating that the tumor behavior could be influenced by both: tumor and microenvironment [9, 10]. The role of galectin-3 in the host tissue modulating the tumor biology is not completely understood [11, 12]. Although the deletion of galectin-3 [13] does not cause any developmental defect, it affects the inflammatory response by modifying the cell mobilization, differentiation and the fibrotic tissue reactions in several pathological conditions [14–16]. In addition, the galectin-3-deficient mice produce lower levels of inflammatory cytokines in draining lymph nodes and, present structural and functional differences in the bone marrow and lymph nodes, that could be relevant in the dissemination of the tumor cells [17, 18].

Although galectin-3 modulates important functions in immunocompetent and inflammatory cells [17–19], its role in tissues involved with tumor dissemination as lymph nodes and hematopoietic bone marrow is poorly explored. Previous studies using intravenous injection of B16F1 melanoma cells in Lgals3−/− mice, have demonstrated an attenuation of metastatic spread in lung of these mice compared with those without deletion of galectin-3 [19]. In our study, we used an orthotopic 4T1 breast cancer model established in Lgals3−/− mice as a suitable experimental animal model to study the role of host galectin-3 in primary tumor growth and metastatic spread. Our results demonstrate that the absence of host galectin-3 confers a selective growth advantage to tumor cells, facilitating the metastatic spread of cancer cells to the lymph nodes and bone marrow. In addition, we also found a differential distribution pattern of CXCL12 in the bone marrow of healthy Lgals3−/− control mice, which may contribute for preparing a much more favorable pre-metastatic niche for further metastasis.

## Methods

### Animals

Eight- to 12-week-old female Lgals3+/+ or Lgals3−/− Balb/c mice [20] were obtained from the animal facilities of the Medical School of the University of São Paulo (USP) and used in all experiments. All animal experiments were in compliance with the relevant laws and were approved by the Ethics Committee of Animal Use of the Federal University of Rio de Janeiro (registration number: DAHEICB069).

### Breast cancer cell line

Balb/c mouse breast cancer cell line 4T1 was a donation from Dra. Adriana Bonomo (Oswaldo Cruz Institute - FIOCRUZ), Rio de Janeiro, Brazil and maintained in RPMI supplemented with 10 % of FBS. Cells were routinely maintained in under confluence monolayers every 3 days and not kept in culture for more than five passages.

### Experimental assay for primary tumor growth and spontaneous metastasis

Balb/c Lgals3+/+ and Lgals3−/− female mice were inoculated in the fourth mammary fat pad with 105 cells in 100 μl [21]. The tumor size was evaluated macroscopically, by measuring the external diameter and weight of the excised tumor. The maximum diameter of the primary tumors was obtained by ultrasound measurement as described by Suzuki et al. [22].

### Histological analysis of primary tumor, draining lymph nodes and iliac crest bone marrow

After 21 and 28 days post orthotopic 4T1 injections the primary tumor and draining lymph nodes, were collected, cleaved and fixed in 4 % PFA. The paraffin-embedded tissues were staining for H&E and proliferative cells were stained using Ki-67. Angiogenesis in the tumor section was evaluated by the immunohistochemistry using the monoclonal antibody anti-CD31 and analyzed in five random fields per tumors and in three primary tumors per group. The lumen was measured using the software Axioplan®. and their lumen’s area was quantified by the software Axioplan®, through the mathematical deconvolution method. The necrotic area was measured using the same software Axioplan®. The iliac crest bone marrow was collected, fixed in paraformaldehyde 4 % buffered solution for 1 day and decalcified in EDTA 20 % for additional 14 days and then embedded in paraffin. Slices of 5 μm were obtained and were stained with H&E, and the immunohistochemistry for CK-19 and Ki-67 antibodies.

### Clonogenic metastatic assay

The draining nodes and iliac crest bones were harvested after 21 and 28 days post-injection of tumors cells and dissociated physically. The cells suspensions were cultured in serial dilution in DMEM medium at FBS 10 % in the presence of 6-thioguanine at concentration of 1 μg/mL. After 14 days in culture, the metastatic cells in LNs and iliac bone marrow of Lgals 3+/+ and Lgals3−/− female mice were stained with methylene blue and quantified by their selection based on the resistance to 6-thioguanine [23].

### RNA extraction, reverse transcription and quantitative PCR

Total RNA from tissue was isolated using the Tri-Reagent (Sigma) according to the manufacturer’s instructions. Complementary DNA (cDNA) was synthesized from 1 μg of total RNA using the High capacity cDNA RT kit (Applied Biosystems), according to the manufacturer’s protocols. Quantitative PCR analysis was performed in triplicate using Power SYBR Green Master Mix (Applied Biosystems). Relative quantification was done using the Ct method normalizing to β-actin gene expression.

**Table:** 

**Primer**	**Forward 5’ – 3’**	**Reverse 5’ – 3’**
β-actin	CTAAGGCCAACCGTGAAAAG	ACCAGAGGCATACAGGGACA
CK-19	TGACCTGGAGATGCAGATTG	CCTCAGGGCAGTAATTTCCTC

### Statistical analyses

Statistical analyses were performed using GraphPad Prism 6.0 software (GraphPad Software, Inc.). Results are shown as means ± standard deviation (S.D.). To determine statistically significant differences between groups, normal distribution was assumed and unpaired Student’s t-test or one-way analysis of variance (ANOVA) were used. For xenograft studies, the growth rates were calculated by non-linear regression (exponential growth model). *P* < 0.05 was considered as statistically significant.

## Results

### Galectin-3 deficiency provides a more permissive environment for the growth of 4T1 carcinoma cells in the mammary fat pad

**Fig. 1:**
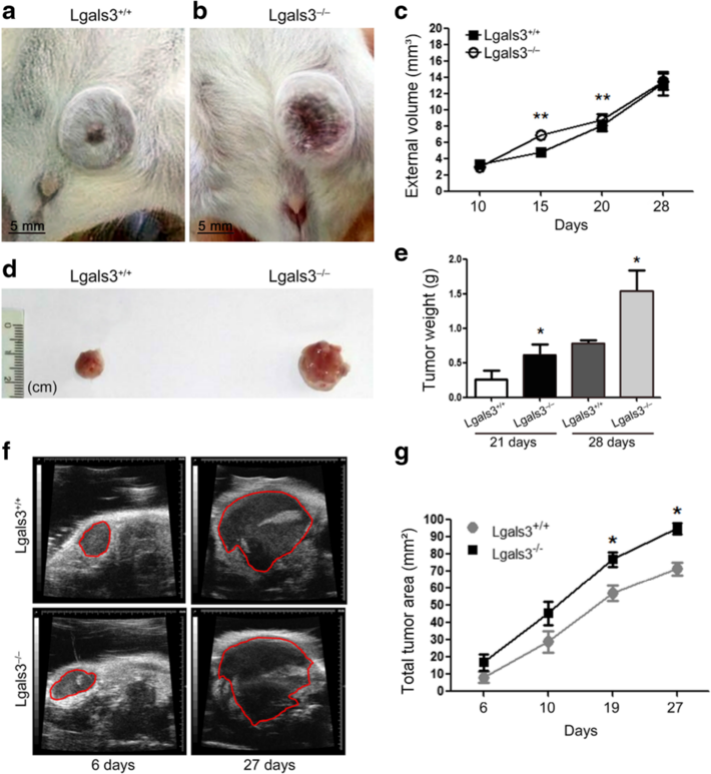
The growth rate of mammary cancer 4T1 is delayed in Lgals3−/− mice. Balb/c Lgals3+/+ and Lgals3−/− females mice were inoculated with 105 4T1 mammary carcinoma cells in the fourth mammary fat pad. Representative image of 4T1 tumor in **a** (Lgals 3+/+) and **b** (Lgals3−/−) mice 28 days p.o.i. **c** Tumor volume of 4T1 tumors in Balb/c Lgals3+/+ and Lgals3−/− mice. **d** Representative image of 4T1 tumor excised from Balb/c Lgals3+/+ and Lgals3−/− mice 28 days p.o.i. **e** Tumor weight of 4T1 tumors in Balb/c Lgals3+/+ and Lgals3−/− mice 21 and 28 days p.o.i. **f** Representative image of 4T1 tumor ultrasonography from Balb/c Lgals3+/+ and Lgals3−/− mice 6 and 27 days p.o.i. **g** Tumor area of 4T1 tumors in Balb/c Lgals3+/+ and Lgals3−/− mice measured by ultrasonography. Data are the mean ± S.D., *n* = 4, three animals per group; *p < 0.05, **p < 0.01

Initially, we evaluated the susceptibility of Lgals3+/+ or Lgals3 −/− female mice to 4T1 cells tumor growth. Ten days post orthotopic injection (p.o.i), 4T1-derived tumors were macroscopically detected in both groups (Fig. 1a and b). However, 15 and 20 days p.o.i we observed an increased tumor volume in Lgals3−/− mice in comparison with Lgals3+/+ (Fig. 1c, *P* < 0.01). We then excised 4T1-derived tumors 21 or 28 days p.o.i. and found that Lgals3−/− derived tumors presented an increased size (Fig. 1d, Additional file 1: Figure S1, Additional file 2) and weight (Fig. 1e, *P* < 0,05) in comparison with Lgals3+/+ − derived tumors. Using ultrasonography (USG) we were able to find an increased growth of 4T1 tumor cells in Lgals3−/− mice (line’s slope = 99,19) in comparison with Lgals3+/+ mice (line’s slope = 91,74). The tumor area was higher in Lgals3−/− mice after 19 days p.o.i. (Fig. 1f and g, *p* < 0,05).

**Fig. 2:**
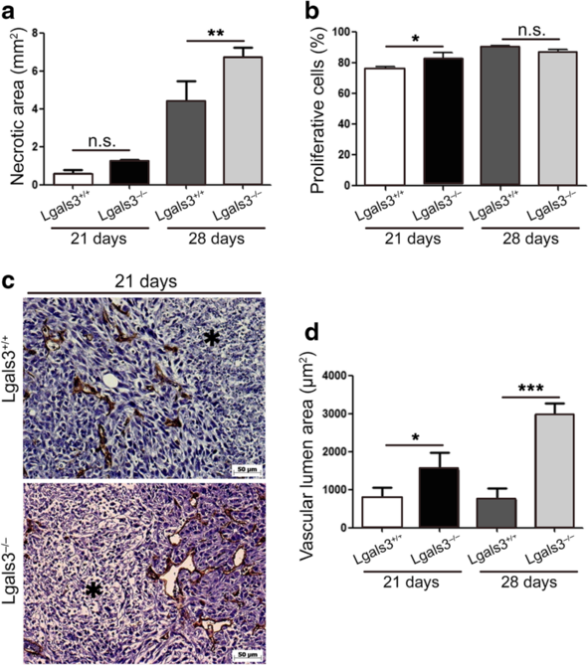
4T1-derived tumors have increased necrotic area, proliferation and blood vessels in Lgals3−/− mice. Quantification of **a** the necrotic area and **b** Ki67 in 4T1 tumors inoculated in Balb/c Lgals3+/+ or Lgals3−/− mice 21 and 28 days p.o.i. **c** Representative immunohistochemical staining of CD31 and (**d**) quantification of the vascular lumen area in 4T1 tumor inoculated in Balb/c Lgals3+/+ or Lgals3−/− mice 21 and 28 days p.o.i. Data are the mean ± S.D., *n* = 4, three animals per group; *p < 0.05, **p < 0.01, ***p < 0.001

We then performed histological analysis of the primary tumor 21 and 28 days p.o.i. and found an increased necrotic area (Fig. 2a, *p* < 0,01) and percentage of proliferative cells (Fig. 2b, *p* < 0,05) in 4T1 tumors grown in the absence of galectin-3 (Lgals3−/−). Although no difference could be observed regarding the number of blood vessels in 4T1-derived tumors, we found a significant increase in the vessel lumen area of tumors grown in Lgals3−/− mice (Fig. 2c and 2d, *p* < 0,05). Altogether, these data demonstrate that the absence of galectin-3 in the host confers a selective growth advantage for tumor in the primary site.

### Galectin-3 deficiency favors the metastatic spread of 4T1 carcinoma cells to the draining lymph nodes

**Fig. 3:**
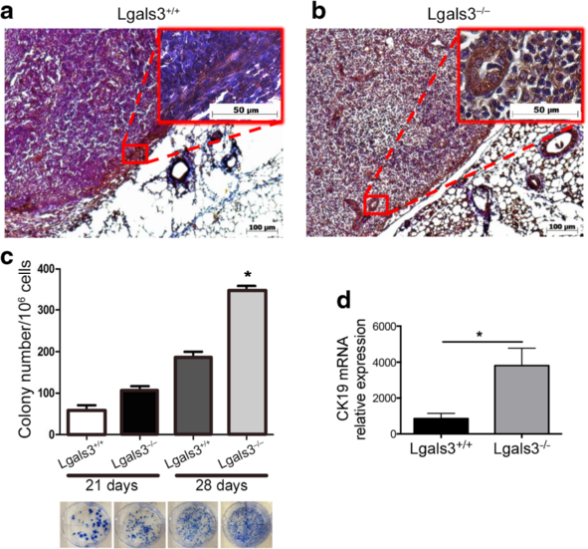
The detection of 4T1-derived metastatic colonies in the lymph nodes is increased in Lgals3−/− mice. Representative immunohistochemical staining of CK-19 in the draining lymph nodes of Balb/c **a** Lgals3+/+ or **b** Lgals3−/− mice previously inoculated with 105 4T1 mammary carcinoma cells in the fourth mammary fat pad for 28 days. **c** Number and representative images of clonogenic 4T1 metastatic cells cultured from a total of 105 draining lymph nodes cells 21 and 28 days p.o.i. **d** CK-19 mRNA levels in draining lymph nodes cells of Balb/c Lgals3+/+ or Lgals3−/− mice 15 days p.o.i. with 4T1 mammary carcinoma cells. Data are the mean ± S.D., *n* = 4, three animals per group; *p < 0.05

We next investigated whether galectin-3 could influence the development of metastasis to the lymph node. Therefore, 28 days post orthotopic injection (p.o.i) of 4T1 cells in Lgals3+/+ or Lgals3−/− mice, the lymph nodes were excised and the presence of CK-19 positive cells was analyzed by immunohistochemistry. We observed that 4T1 cells (CK-19+) were predominantly present in the capsule of the draining lymph node in Lgals3+/+ mice (Fig. 3a) whereas in Lgals3−/− mice, CK-19+ cells were organized as “sheets-like” within the lymph node parenchyma and also found in the capsule (Fig. 3b). Moreover, we evaluated the presence of lymph node metastasis in Lgals3+/+ and Lgals3−/− mice using the 6-thioguanine clonogenic assay and found significant fewer metastasis in Lgals3+/+ mice in comparison to Lgals3−/− mice, both 21 and 28 days p.o.i. (Fig. 3c, *p* < 0,05). Interestingly though, we also found an increased CK-19 mRNA levels in Lgals3−/− mice at an earlier stage (15 days) p.o.i. (Fig. 3d, *p* < 0,05). These results suggest that Lgals3−/− mice are more permissive for 4T1 tumor cells dissemination to the inguinal lymph nodes.

### Galectin-3-deficient bone marrow microenvironment supports more efficiently the growth of metastatic 4T1

**Fig. 4:**
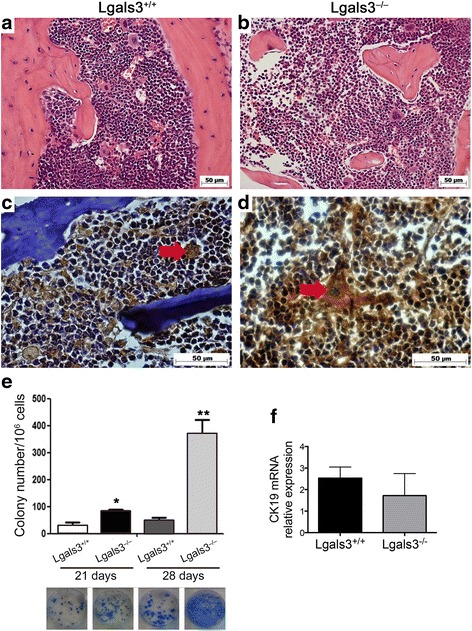
The detection of 4T1-derived metastatic colonies in the bone marrow is increased in Lgals3−/− mice. Representative immunohistochemical staining of hematoxylin and eosin in the bone marrow of Balb/c **a** Lgals3+/+ or **b** Lgals3−/− mice. Representative immunohistochemical staining of CK-19 in the bone marrow of Balb/c c Lgals3+/+ or **d** Lgals3−/− mice previously inoculated with 105 4T1 mammary carcinoma cells in the fourth mammary fat pad for 28 days. **e** Number and representative images of clonogenic 4T1 metastatic cells cultured from a total of 105 iliac bone cells 21 and 28 days p.o.i. **f** CK-19 mRNA levels in iliac bone cells of Balb/c Lgals3+/+ or Lgals3−/− mice 15 days p.o.i. with 4T1 mammary carcinoma cells. Data are the mean ± S.D., *n* = 3, three animals per group; *p < 0.05, **p < 0.01

We have previously described that Lgals3−/− mice presented structural and functional differences in the bone marrow [17]. Likewise, in this study we confirmed differences in terms of cellularity and projections of bone tissue inside the cavity between Balb/c Lgals3+/+ and Lgals3 −/− mice (Fig. 4a and b). 28 days p.o.i of 4T1 cells in Lgals3+/+ or Lgals3−/− mice, we observed that CK-19+ cells were easier visualized in the hematopoietic compartment of the Lgals3−/− female mice compared with the Lgals3+/+ group (Fig. 4c and d, arrow).

In contrast to observed in inguinal lymph nodes, in 15 days p.o.i, no differences in CK-19 m RNA levels was detected in bone marrow (Fig. 4f). When we compared the bone marrow metastasis between the groups by the 6-thioguanine clonogenic assay, we found significant fewer metastasis in Lgals3+/+ mice in comparison to Lgals3−/− mice after 21 and 28 days p.o.i of 4T1 cells. (Fig. 4e, *p* < 0,05). These results indicate that bone marrow compartment of Lgals3−/− mice displays favorable environmental conditions for tumor cell to colonize and survive, after 21 days p.o.i.

### The absence of galectin-3 changes the spatial distribution of CXCL12 in the bone marrow

**Fig. 5:**
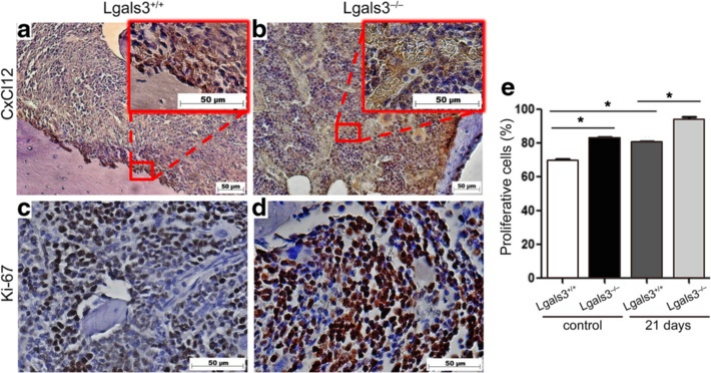
The spatial distribution of CxCl12 is altered in the bone marrow of Lgals3−/− mice. Representative immunohistochemical staining of (**a** and **b**) CxCl12 or (**c** and **d**) Ki-67 in the bone marrow of Balb/c Lgals3+/+ or Lgals3−/− mice. **e** Quantification of Ki-67 in the bone marrow of Balb/c Lgals3+/+ or Lgals3−/− mice. Data are the mean ± S.D., *n* = 4, three animals per group; *p < 0.05

We finally investigated a possible mechanism by which the absence of galectin-3 in the host could favor 4T1 spread to the bone marrow. Since 4T1 cells are CXCR4 positive (Additional file 2, Additional file 3: Figure S2) we next evaluated the protein expression of CXCL12 in the bone marrow of healthy Lgals3+/+ and Lgals3−/− mice by immunohistochemistry. We observed that CXCL12 was predominantly present in the endosteal region of the bone marrow in Lgals3+/+ mice (Fig. 5a). In contrast, CXCL12 was mainly found scattered throughout the bone marrow of Lgals3−/− mice (Fig. 5b). Interestingly, we observed a higher rate of proliferative cells in the bone marrow of Lgals3−/− mice in comparison with Lgals3+/+ mice (Fig. 5c and d) both in control and in 21 days p.o.i of 4T1 cells (Fig. 5e, *p* < 0,05). In 28 days p.o.i. the rate of proliferative cells was not statistically significant. These results suggest that the differential distribution of CXCL12 found in Lgals3−/− mice may provide a more favorable niche for incoming tumor cells to proliferate in the hematopoietic bone marrow.

## Discussion

In the past few decades considerable progresses have been made to understand the molecular basis of metastasis. However, the underlying events of tumor metastasis are still not well understood. In this study we demonstrated that tumors derived from Lgals3−/− mice orthotopically injected with 4T1 cells displayed: (1) a higher proliferative rate, an increased necrotic area and new blood vessels with a wider lumen; (2) higher metastatic colonies in the lymph nodes and the bone marrow. (3) Moreover, we found a different spatial distribution of CXCL12 in the bone marrow of Lgals3−/− mice, which could contribute for the increase colonization of 4T1 cells to this organ.

Our data demonstrated that the absence of host galectin-3 drastically affected the tumor biology. So far, few studies have addressed the role of host galectin-3 in carcinogenesis and metastasis. A study using B16F1 melanoma cells, a variant of B16 melanoma possessing lower metastatic potential than B16F10 cells, demonstrated that C57/BL6 Lgals3−/− mice were more competent in terms of their anti-tumor immunity when compared to Lgals3+/+ mice and, presented enhanced NK-cell activity and lower metastasis [19, 24]. In contrast, More SK, 2015 [25] found that C57/BL6 Lgals3−/− mice showed similar extent of B16F10 melanoma metastatic colonies in the lung as the Lgals3+/+ mice. Another group described that Lgals3−/− mice facilitated B16F10 lung metastasis as a result of decreased NK cytotoxicity and disturbed serum Th1, Th2 and Th17 cytokines [26]. On the other hand, a study using B16F10 melanoma cell and LLC lung cancer cells in an allograft model and found an increased primary solid tumor growth in C57/BL6 Lgals3−/− mice compared with Lgals3+/+ mice in both B16 and LLC tumors [27]. In our study we orthotopically injected 4T1 tumor cells in Balb/c Lgals3−/− and Lgals3+/+ mice strain. To our best knowledge this is the first study that mimics human breast cancer in a galectin-3 depleted environment model and closely simulate human cancer progression and metastasis.

Our study is not entirely free of galectin-3 since 4T1 tumor cells express galectin-3, which is believed to confer survival advantage to tumor cells during dissemination. Several reports have demonstrated that galectin-3 interaction with its glycoconjugate ligands increased cancer homotypic aggregation to form a tumor micro-emboli and cancer cell heterotypic adhesion to the blood vascular endothelium [28–31]. Moreover, the levels of circulating galectin-3 in the bloodstream of patients with metastasis are significantly higher than those of healthy people [6]. In addition, both intra and extracellular galectin-3 suppress apoptosis induced by the loss of cell anchorage (anoikis) [7, 29]. Therefore, in our study, galectin-3 expression by tumor cells may have enhanced the survival of disseminating tumor cells in the circulation.

The host tissue microenvironment plays a key role for tumor cells colonization of secondary organs. Our data indicate that loss of galectin-3 makes the lymph node and the bone marrow a favorable microenvironment for metastatic colonization. Although Lgals3−/− mice are viable and fertile, these animals present multiple disorders associated with inflammation and immune response [3, 14, 26, 20, 32–35] and endochondral ossification [31]. Therefore the absence of host galectin-3 may lead to a decreased immune response against the tumor and be an important predisposing factor for tumor growth in the primary site and for the dissemination of tumor cells to the lymph nodes and bone marrow compartment.

We have previously shown that Lgals3−/− mice presented a reduced cell density and diaphyseal disorders with functional differences in the bone marrow cavity in comparison to Lgals3+/+ mice [17]. Here we found differences in terms of cellularity and projections of the bone tissue between both groups that could also explain the increased dissemination of 4T1 cells in Lgals3−/− mice to the bone marrow.

The bone marrow compartment is a dynamic environment constituted by a rich milieu of growth factors, stromal cells and a complex extracellular matrix network necessary to maintain homeostasis of the hematopoietic system. The equilibrium between proliferation and differentiation of the hematopoietic stem cells (HSC) is controlled by endosteal region, a site of HSC niche, maintained mainly by the attractive chemokine (CXCL12) and by a central region, responsible for generation of different hematopoietic progenitor cells [36] Based on these characteristics, the bone marrow microenvironment is a fertile soil not only for HSC and its progenies, but also or the growth of cancer cells. It is well described that the several solid tumors, including breast, ovarian and prostate migrate to bone marrow compartment by the same mechanism used by normal hematopoietic stem cell, the CXCL12-CXCR4 axis. The CXCL12 is an attractive chemokines produced constitutively by stromal cells and its interaction with the ligand controls the cell retention inside the bone marrow [37] The attractive chemokines expressed by the stromal bone marrow cells can also stimulate the survival of malignant cells causing them to growth in the hematopoietic stem cell niches in the endosteal region [38]. In a recent report [39], supported the hypothesis that CXCL12-CXCr4 axis promotes the natural selection of breast cancer cell metastasis with implications for tumor aggressiveness. Moreover, the presence of CXCL12 in a non-canonical region of the bone marrow, could amplify the CXCL12-CXCR4 axis, favoring the proliferation of cancer cells. Since the localization of CXCL12 chemokine is strongly modified by galectin-3 deletion and is detected in the overall area of the bone marrow, it may facilitate the attraction, maintenance and survival of 4T1 cells in Lgals3−/− mice in comparison with a normal microenvironment.

## Conclusions

Our data demonstrated that the absence of host galectin-3 drastically affected the tumor biology favoring the metastatic spreading of 4T1 cells to inguinal lymph nodes and bone marrow colonization.

## Additional files

Additional file 1: Figure S1. Immunohisotchemistry to localize Galectin-3 in primary tumor and in peri-tumoral area Lgals-3+/+ and Lgals-3−/− female mice. (A and C) Tumor of Lgals-3+/+ of after 21 and 28 days p.o.i. (B and D) Tumor of Lgals-3−/− of after 21 and 28 days p.o.i. (E) quantification of galectin-3 positive cells in primary tumor and in peri-tumoral tissue (*). Data are the mean ± S.D., n=4, three animals per group; *** p<0.001. (TIF 2731 kb) Additional file 2: Supplemental methods. (PDF 316 kb) Additional file 3: Figure S2. 4T1 cells express galectin-3, CK-19 and CXCR4 proteins. Representative immunocytochemical staining of (a) galectin-3 (b) CK-19 and (c) CXCR4. The negative control of each reaction is represented in the figures (*). (TIF 6055 kb)

## Abbreviations

CK-19: Cytokeratin 19
CRD: C-terminal Carbohydrate Recognition Domain
Lgals3−/−: Galectin-3 Knockout Mice
Lgals3+/+: Wild Type Mice
p.o.i: Post Orthotopic Injection
USG: Ultrasonography
