# Supplementary figures and images for: The deficiency of galectin-3 in stromal cells leads to enhanced tumor growth and bone marrow metastasis
Source: BMC Cancer. 2016 Aug 15;16:636. doi: 10.1186/s12885-016-2679-1 (PMC4986277; doi:10.1186/s12885-016-2679-1)

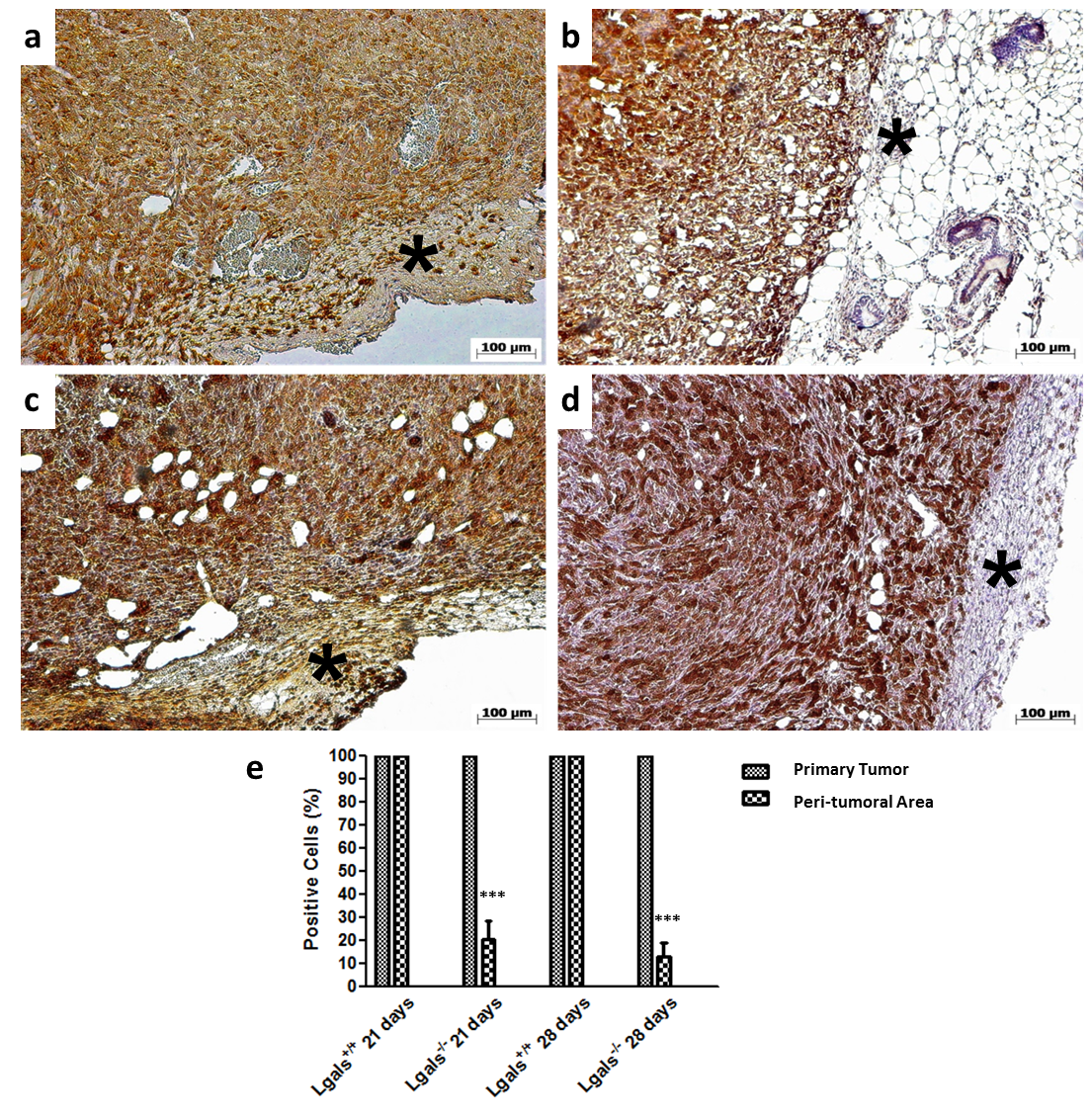

Supplement: Additional file 1: Figure S1. — Immunohisotchemistry to localize Galectin-3 in primary tumor and in peri-tumoral area Lgals-3+/+ and Lgals-3−/− female mice. (A and C) Tumor of Lgals-3+/+ of after 21 and 28 days p.o.i. (B and D) Tumor of Lgals-3−/− of after 21 and 28 days p.o.i. (E) quantification of galectin-3 positive cells in primary tumor and in peri-tumoral tissue (*). Data are the mean ± S.D., n=4, three animals per group; *** p<0.001. (TIF 2731 kb) [file 12885_2016_2679_MOESM1_ESM.tif]

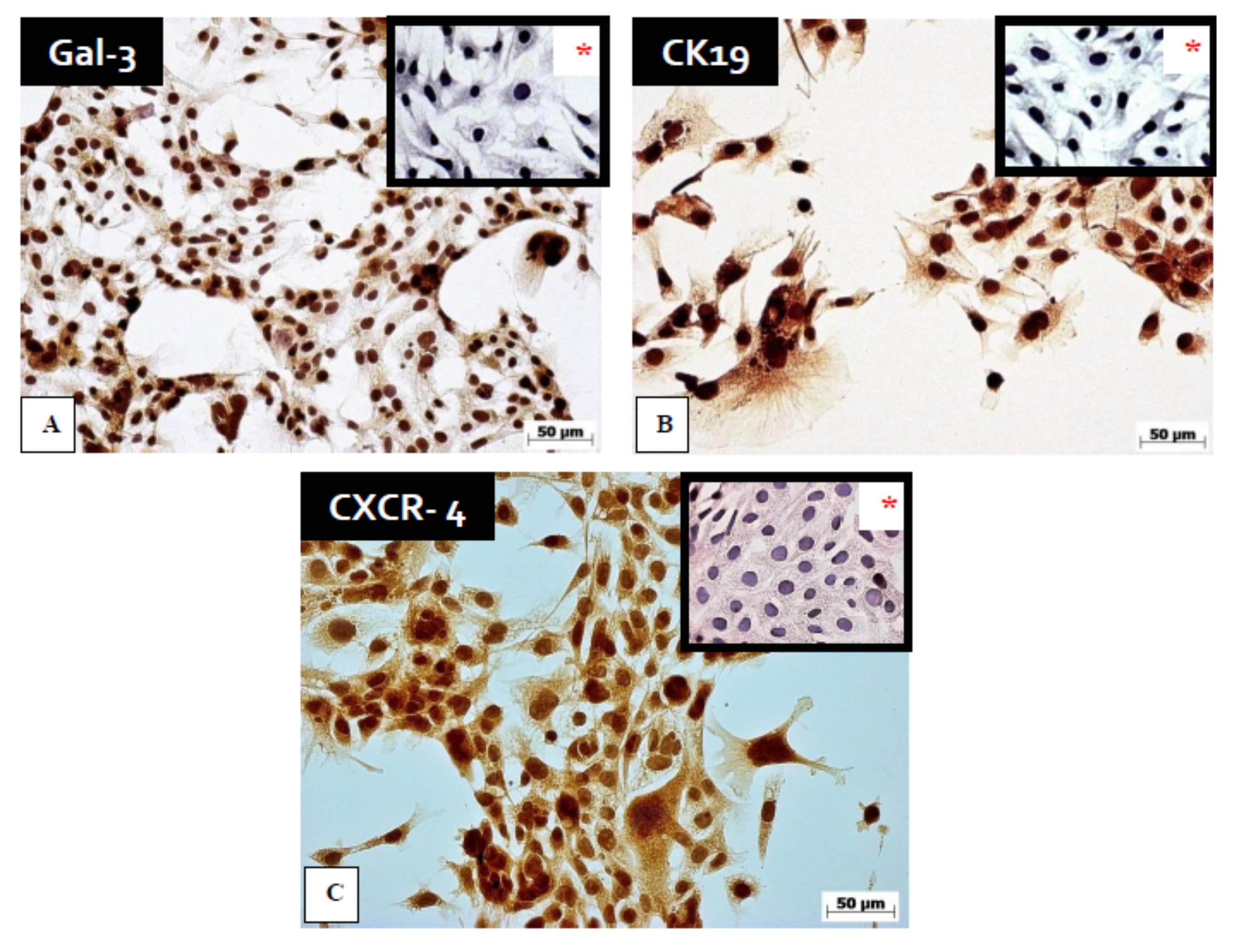

Supplement: Additional file 3: Figure S2. — 4T1 cells express galectin-3, CK-19 and CXCR4 proteins. Representative immunocytochemical staining of (a) galectin-3 (b) CK-19 and (c) CXCR4. The negative control of each reaction is represented in the figures (*). (TIF 6055 kb) [file 12885_2016_2679_MOESM3_ESM.tif]
