# Supplementary material for: The deficiency of galectin-3 in stromal cells leads to enhanced tumor growth and bone marrow metastasis
Source: BMC Cancer. 2016 Aug 15;16:636. doi: 10.1186/s12885-016-2679-1 (PMC4986277; doi:10.1186/s12885-016-2679-1)
Supplement: Additional file 2: — Supplemental methods. (PDF 316 kb) [file 12885_2016_2679_MOESM2_ESM.pdf]

The deficiency of galectin-3 in stromal cells leads to enhanced tumor growth and bone marrow metastasis

Jonathas Xavier Pereira[1], Maria Carolina Braga de Azeredo[2], Felipe Sá Martins[3], Roger Chammas[4], Felipe Leite de Oliveira[5], Sofia Nascimento dos Santos[6], Emerson Soares Bernardes[7] and Márcia Cury El Cheikh[8].

1. Pereira, JX. Programa de Pós-Graduação em Anatomia Patológica, Hospital Clementino Fraga Filho, UFRJ, Rio de Janeiro, Brasil. E-mail: jonathasxp@gmail.com
2. Azeredo, MCB. Programa de Pós-Graduação em Ciências Morfológicas, ICB, UFRJ, Rio de Janeiro, Brasil. E-mail: mcbazeredo@yahoo.com.br
3. Martins, FS. Universidade Federal do Rio de Janeiro, Rio de Janeiro, Brasil. E-mail: felipedesa.farma@gmail.com
4. Chammas, R. Laboratório de Oncologia Experimental e Instituto do Câncer do Estado de São Paulo, Faculdade de Medicina, São Paulo, Brasil. E-mail: rchammas@usp.br
5. Oliveira, FL. Laboratório de Proliferação e Diferenciação Celular, ICB. UFRJ, Brasil. E-mail: felipe@histo.ufrj.br
6. Santos, SN. Centro de Radiofarmácia, Instituto de Pesquisas Energéticas e Nucleares (IPEN), São Paulo, Brasil. E-mail: snsantos85@gmail.com
7. Bernardes, ES. Centro de Radiofarmácia, Instituto de Pesquisas Energéticas e Nucleares (IPEN), São Paulo, Brasil. E-mail: emerson.bernardes@gmail.com
8. El-Cheikh, MC (*). Laboratório de Proliferação e Diferenciação Celular, ICB. UFRJ, Brasil. E-mail: marcia@histo.ufrj.br

1 and 2, both contributed equally for the work

(*) Corresponding author. Av. Carlos Chagas Filho, 393. Bloco F. CEP. 21941-902. Cidade Universitária, Ilha do Fundão, Instituto de Ciências Biomédicas, CCS, Rio Janeiro, RJ, Brasil. e-mail: marcia@histo.ufrj.br.

Additional file 3

Additional file Figures legend

Additional file 1: Figure S1. Immunohisotchemistry to localize Galectin-3 in primary tumor and in peri-tumoral area Lgals-3+/+ and Lgals-3-/- female mice. (A and C) Tumor of Lgals-3+/+ of after 21 and 28 days p.o.i. (B and D) Tumor of Lgals-3-/- of after 21 and 28 days p.o.i. (E) quantification of galectin-3 positive cells in primary tumor and in peri-tumoral tissue (*). Data are the mean ± S.D., n=4, three animals per group; *** $p<0.001$.

Additional file 2: Figure S2. 4T1 cells express galectin-3, CK-19 and CXCR4 proteins. Representative immunocytochemical staining of (**a**) galectin-3 (**b**) CK-19 and (**c**) CXCR4**.** The negative control of each reaction is represented in the figures (*).

Supplemental methods

Supplemental methods 1

Immunohistochemistry in the primary tumors.

Primary tumors were fixed in 4% paraformaldehyde and stained with anti-galectin-3 (M3/38 monoclonal antibody, ATCC, USA) followed by a secondary anti-rat biotinylated antibodies. Next, avidin-peroxidase (Sigma-Aldrich, USA) was added and color development was done with diaminobendizine. The positive cells were quantified in three random fields inside and in peri-tumoral area by TMarker software.

Supplemental methods 2

Immunocytochemistry of 4T1 cells

4T1 cells were fixed in 4% paraformaldehyde and stained with anti-CK-19 (purified polyclonal antibody – Abcam), anti-galectin-3 (M3/M8 polyclonal antibody, ATCC, USA) and anti-CXCR4 (purified polyclonal antibody – Abcam) followed by a secondary anti-rabbit or anti-rat biotinylated antibodies. Next, avidin-peroxidase (Sigma-Aldrich, USA) was added and color development was done with diaminobendizine. As a negative control, the same procedure was used without the primary antibody.
